# Therapeutic effects of isoquercetin on ovariectomy-induced osteoporosis in mice

**DOI:** 10.1007/s13659-023-00383-2

**Published:** 2023-06-08

**Authors:** Mengjing Wu, Mengyu Qin, Xian Wang

**Affiliations:** Laboratory of Analytical Chemistry of the State Ethnic Affairs Commission, School of Chemistry and Materials Science, South-Central Minzu University, Wuhan, 430074 China

**Keywords:** Osteoporosis, Isoquercetin, Bone marrow mesenchymal stem cells, Osteogenic induction, Adipogenic induction

## Abstract

Bone marrow mesenchymal stem cells (BMSCs) are non-hematopoietic multipotent stem cells capable of differentiating into mature cells. Isoquercetin, an extract from natural sources, has shown promise as a potential treatment for osteoporosis. To investigate the therapeutic effects of isoquercetin on osteoporosis, bone marrow mesenchymal stem cells (BMSCs) were cultured in vitro, and osteogenesis or adipogenesis was induced in the presence of isoquercetin for 14 days. We evaluated cell viability, osteogenic and adipogenic differentiation, as well as mRNA expression levels of *Runx2, Alpl*, and *OCN* in osteoblasts, and mRNA expression levels of *Pparγ*, *Fabp4*, and *Cebpα* in adipocytes. The results showed that isoquercetin dose-dependently increased cell viability and promoted osteogenic differentiation, as evidenced by Alizarin Red and alkaline phosphatase staining and mRNA expression levels of *Runx2*, *Alpl*, and *OCN* in osteoblasts (*P* < *0.05*). In contrast, isoquercetin inhibited adipogenic differentiation and decreased the mRNA expression levels of *Pparγ, Fabp4,* and *Cebpα* in adipocytes (*P* < *0.05*). In vivo, isoquercetin treatment increased bone quantity and density in an osteoporosis model mice group, as determined by μCT scanning and immunohistochemistry (*P* < *0.05*). These findings suggest that isoquercetin may have therapeutic potential for osteoporosis by promoting the proliferation and differentiation of BMSCs towards osteoblasts while inhibiting adipogenic differentiation.

**Graphical Abstract:**

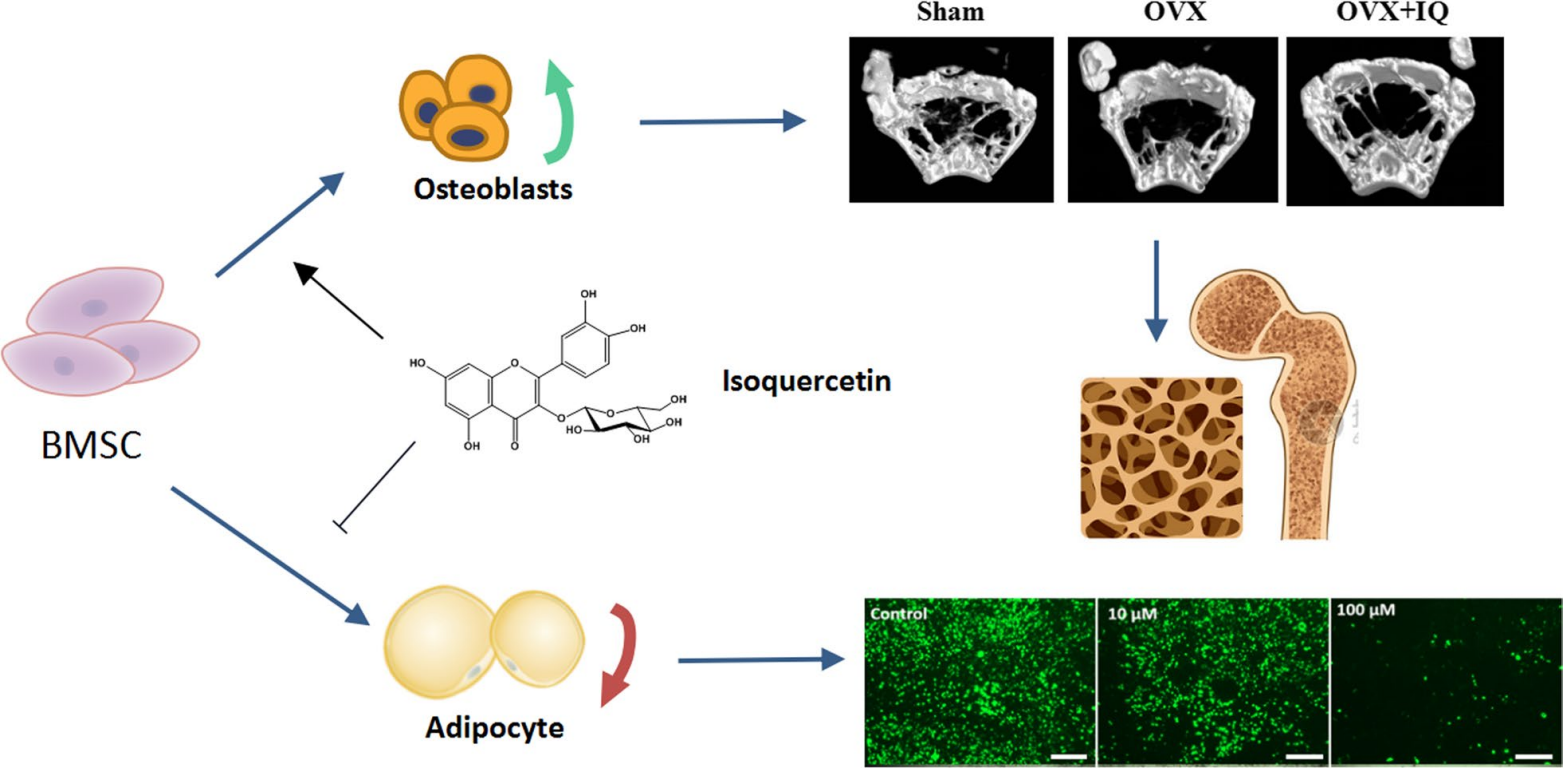

## Introduction

Postmenopausal osteoporosis (PMO) is a metabolic bone disease commonly found in elderly women [1, 2]. It occurs due to the disruption of bone metabolism caused by the decline in ovarian function and the decrease in estrogen levels after menopause [3]. PMO is characterized by decreased bone density, thinner cortical bone thickness, and larger trabecular bone pore size [4]. Estrogen has a direct impact on bone cells, and osteoblasts, osteoclasts, and bone cells are all receptors that express estrogen function [5, 6]. The regulation of bone metabolism homeostasis is primarily controlled by osteoblasts that mediate bone formation and osteoclasts that mediate bone absorption [7]. Osteoblasts (OBs) differentiate from bone marrow mesenchymal stem cells (BMSCs), which can also differentiate into fat cells [8, 9]. During osteoporosis, the number and activity of osteoblasts decrease, and more BMSCs differentiate into fat cells, which is one of the reasons that lead to postmenopausal osteoporosis.

Currently, clinical anti-osteoporosis drugs such as bisphosphonates can effectively inhibit the formation of osteoclasts, but long-term use has limitations and side effects [10, 11]. Bisphosphonates, including alendronate [12], ibandronate [13], and zoledronic acid [14] etc., have been commonly used in the clinical treatment of osteoporosis as they effectively reduce the risk of non-vertebral fractures. However, these drugs have side effects, such as an increased risk of breast cancer, myocardial ischemia, thromboembolic disease, and atypical femoral fractures [15]. Therefore, finding natural anti-osteoporosis drugs has become one of the important research directions.

Dietary plants containing phytoestrogens have been recognized as a possible choice for preventing osteoporosis [16, 17]. In animal models, green tea extract (flavonoids sub-class), such as quercetin, which is rich in flavonoids, has been reported to mitigate the harmful effects of estrogen deficiency on bone loss [18–20]. Quercetin-3-O-β-d-glucoside (isoquercetin) is one of the main glycoside forms of the natural flavonoid quercetin [12]. As the most abundant natural flavonoid, isoquercetin is widely distributed in the plant kingdom and is commonly found in vegetables, fruits, and grains. Isoquercetin has high bioavailability and has a variety of protective effects against oxidative stress, diabetes, cardiovascular disease, cancer, and allergic reactions in vitro and in vivo. In addition, it has a dual-promoting effect on bone metabolism, promoting mesenchymal stem cell differentiation into osteoblasts and reducing osteoclast differentiation. Antioxidation is one of the main pharmacological activities of isoquercetin. Consistently, previous studies reported that IQ can enhance the mineral formation of bone in SaOS-2 cells by up-regulating the expression of two Runx2 cofactors [21]. Dietary IQ exhibited a crucial role in anti-osteoporotic activities by inhibiting hypoxia inducible factor-1 alpha (HIF-1α) and decreasing osteoclasts levels [22].

To investigate the mechanism by which isoquercetin affects osteoblasts, two in vitro cell models were used in this chapter. The first involved extracting primary pre-osteoblasts from the calvaria of neonatal mice and inducing them to mature into osteoblasts to study the effects of isoquercetin on cell proliferation, differentiation, and mineralization. The second model utilized mesenchymal stem cells C3H10T1/2 induced to differentiate into osteoblasts and adipocytes, collectively exploring the cellular-level mechanisms by which isoquercetin acts on osteoblasts.

## Result and discussion

### Isoquercetin for C3H10T1/2 cells activity

**Fig. 1:**
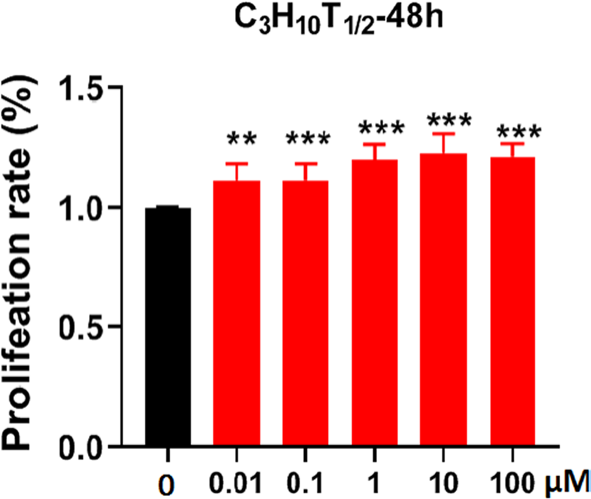
Cell viability assays of C3H10T1/2 cells treated with varying concentrations of isoquercetin (n = 6). Data are shown as mean ± SD *p < 0.05, **p < 0.01, and ***p < 0.001. *P* values were obtained by one-way ANOVA with multiple comparisons and two-tailed Student’s t-test

The CCK-8 assay kit was used to evaluate the effect of isoquercetin on the proliferation of C3H10T1/2 cells. C3H10T1/2 cells were treated with different concentrations of isoquercetin (0.01, 0.1, 1, 10, 100 μM) for 48 h, and compared to the control group, it was found that different concentrations of isoquercetin could promote cell proliferation, with a gradient dependence observed between 0.01 and 10 μM (Fig. 1). The differences between the isoquercetin-treated groups and the control group were statistically significant (*P* < *0.05*). Subsequent cell induction experiments were carried out using isoquercetin at concentrations of 0.1, 1, and 10 μM.

### Results of osteogenesis induction in C3H10T1/2 cells

**Fig. 2:**
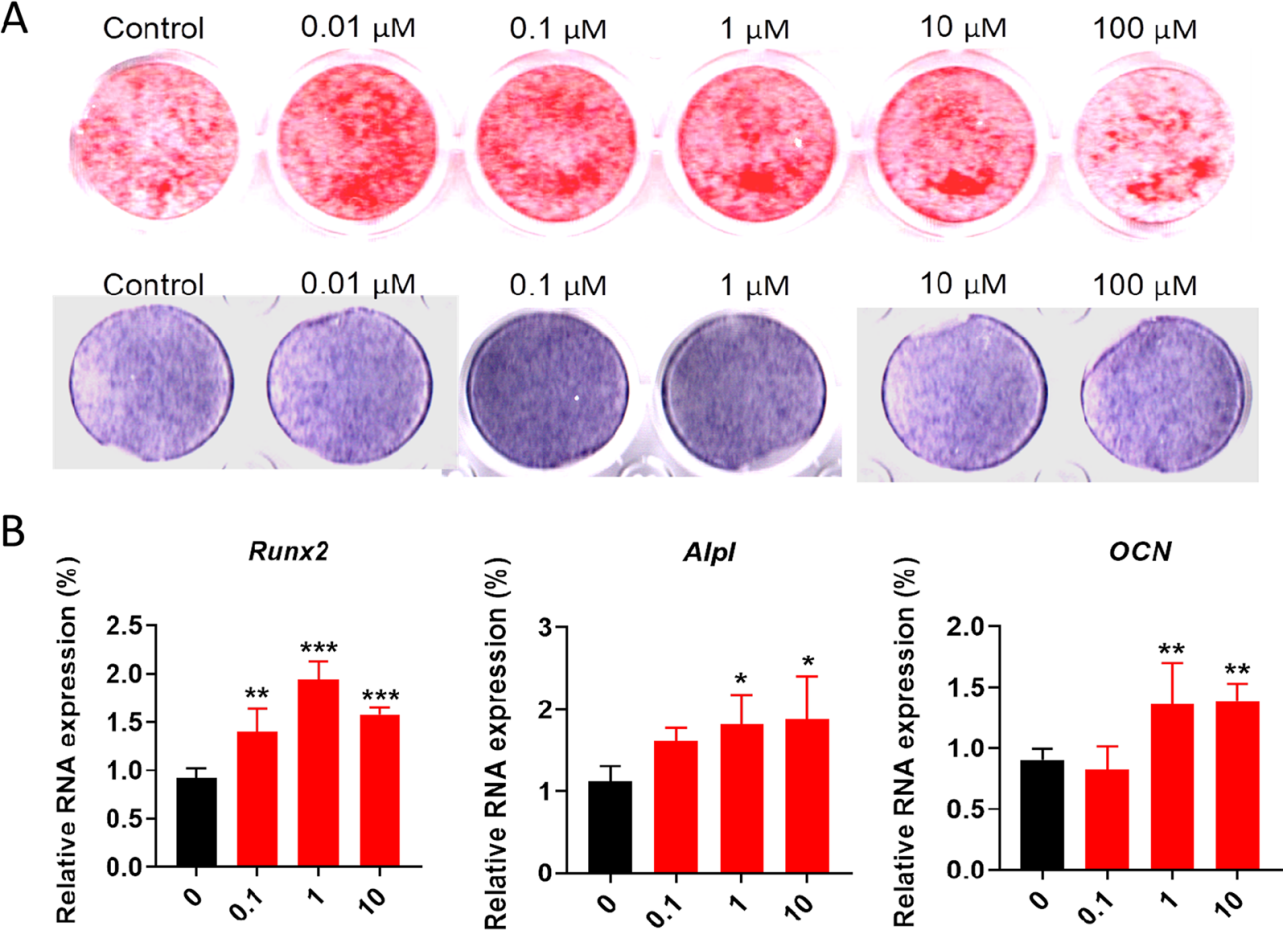
Effect of isoquercetin on the osteogenic differentiation capacity of C3H10T1/2 cells. **A** Alizarin red staining and ALP staining for the OBs treated with isoquercetin. **B** mRNA levels of osteoblast marker (*Alpl* and *Runx2, OCN*) in OBs. Data are shown as mean ± SD. *p < 0.05, **p < 0.01, and ***p < 0.001. *P* values were obtained by one-way ANOVA with multiple comparisons and two-tailed Student’s t-test

A 14-day osteogenic induction experiment was conducted using different gradient concentrations of isoquercetin (0.1, 1, 10 μM) on C3H10T1/2 mesenchymal stem cells. On the 14th day, ALP staining was performed, and compared to the control group, the addition of isoquercetin enhanced ALP activity, with a dependence on color intensity observed. The staining results are shown in Fig. 2A. After 14 days of osteogenic induction, Alizarin Red staining was performed. The staining results showed a positive correlation between the number and area of calcium nodules and the concentration of isoquercetin, with a dependence on color intensity observed (Fig. 2A). In addition, the qPCR results showed that isoquercetin could promote the upregulation of mRNA levels of osteogenic markers *Alpl* and *Runx2*, *OCN*, as shown in Fig. 2B.

### Results of the adipogenic induction in C3H10T1/2 cells

**Fig. 3:**
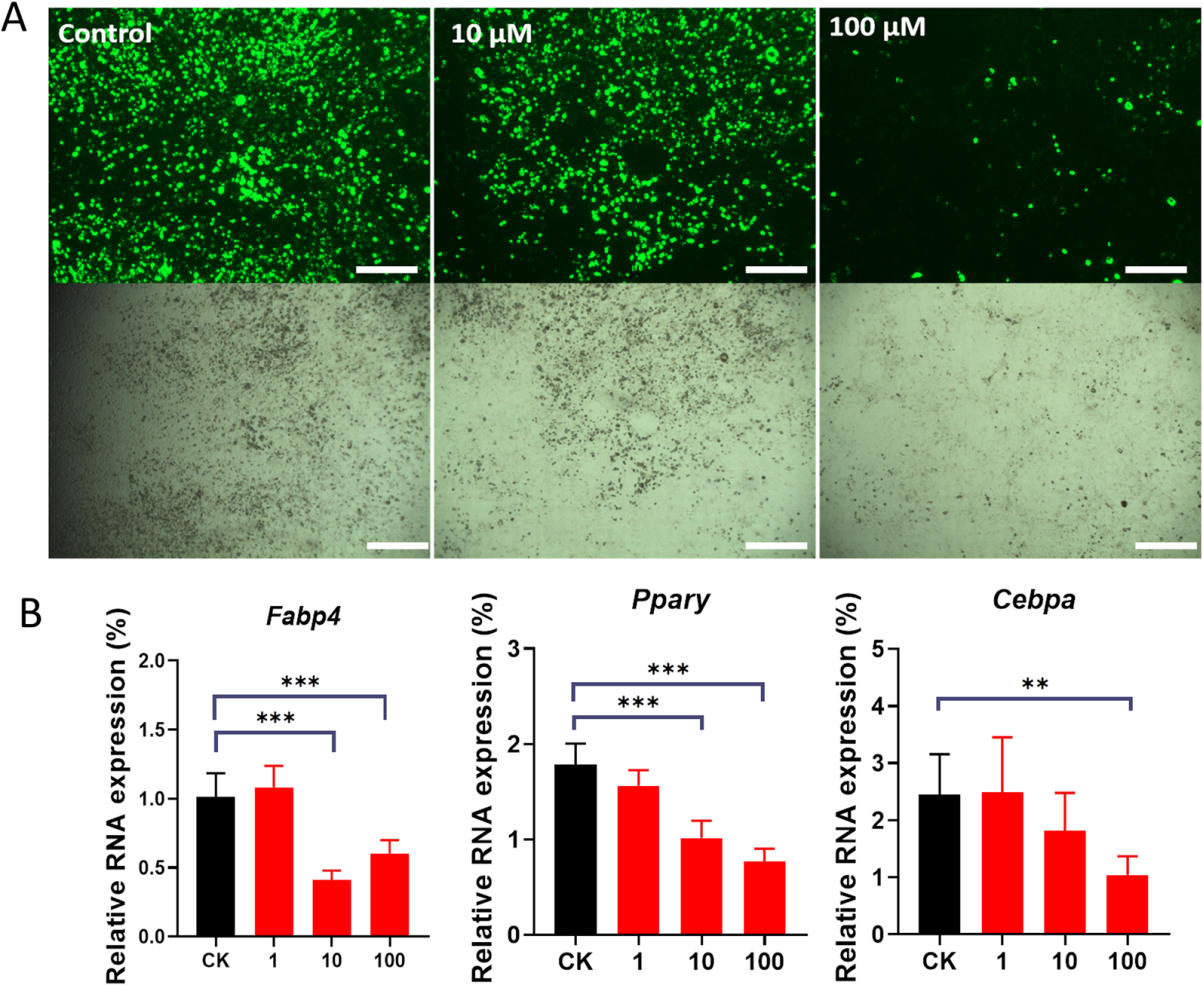
Effect of isoquercetin on the adipogenic differentiation ability of C3H10T1/2 cells. **A** Nile Red fluorescence staining results of adipocytes. (bar = 20 μm). **B** mRNA levels of adipocyte markers (*Pparγ, Fabp4, Cebpα*). Data are shown as mean ± SD. *p < 0.05, **p < 0.01, and ***p < 0.001. *P* values were obtained by one-way ANOVA with multiple comparisons and two-tailed Student’s t-test

Mesenchymal stem cells undergo strict transcriptional regulation towards osteogenic or adipogenic differentiation. In osteoporosis, the differentiation of mesenchymal stem cells towards osteogenic differentiation is weakened, and more towards adipogenic differentiation. Therefore, it is necessary to further explore the effect of isoquercetin on adipogenic differentiation. After inducing C3H10T1/2 adipogenic differentiation for 14 days, Nile red staining was performed on adipocytes and observed under a fluorescence microscope. Under tenfold fluorescence microscopy observation, the control group had more adipocytes, and more fluorescently stained adipocytes, while after dietary intake of isoquercetin, the number of adipocytes decreased, and the fluorescence intensity weakened (Fig. 3A). Nile red fluorescence staining of adipocytes showed that isoquercetin could inhibit mesenchymal stem cells from differentiating toward adipocytes. This study further detected changes in the expression levels of adipogenic differentiation marker genes *Pparγ*, *Fabp4*, and *Cebpa* by qPCR (Fig. 3B). Compared with the control group, it was found that isoquercetin can significantly inhibit the upregulation of adipogenic differentiation markers, and the results are statistically significant (*P* < *0.05)*.

### Isoquercetin results for the improvement of bone mineral density in mice

**Fig. 4:**
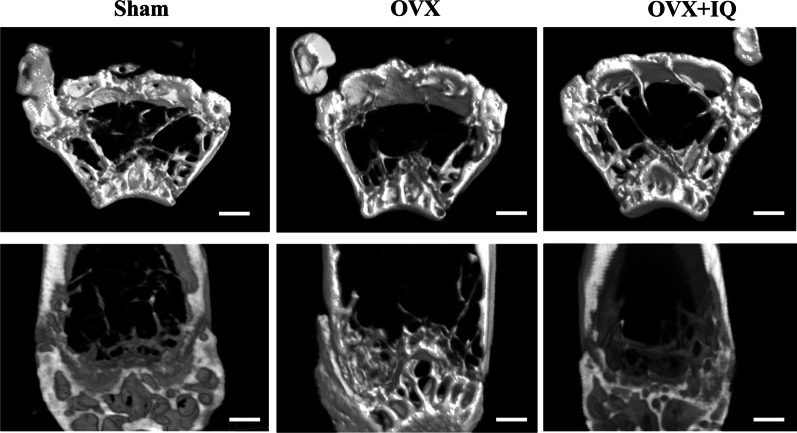
μCT imaging and 3D reconstruction of trabecular bone (Scale bar = 250 μm)

After euthanasia of the mice, three-dimensional reconstruction of the trabecular bone and coronal sections were directly observed through ex vivo μCT scanning. The μCT scan results showed a significant decrease in the trabecular bone in the reconstructed image and coronal section of the OVX group compared to the control group. However, the administration of isoquercetin significantly increased bone density compared to the OVX group, as shown in Fig. 4.

### Morphometric analysis of the bone tissue

**Fig. 5:**
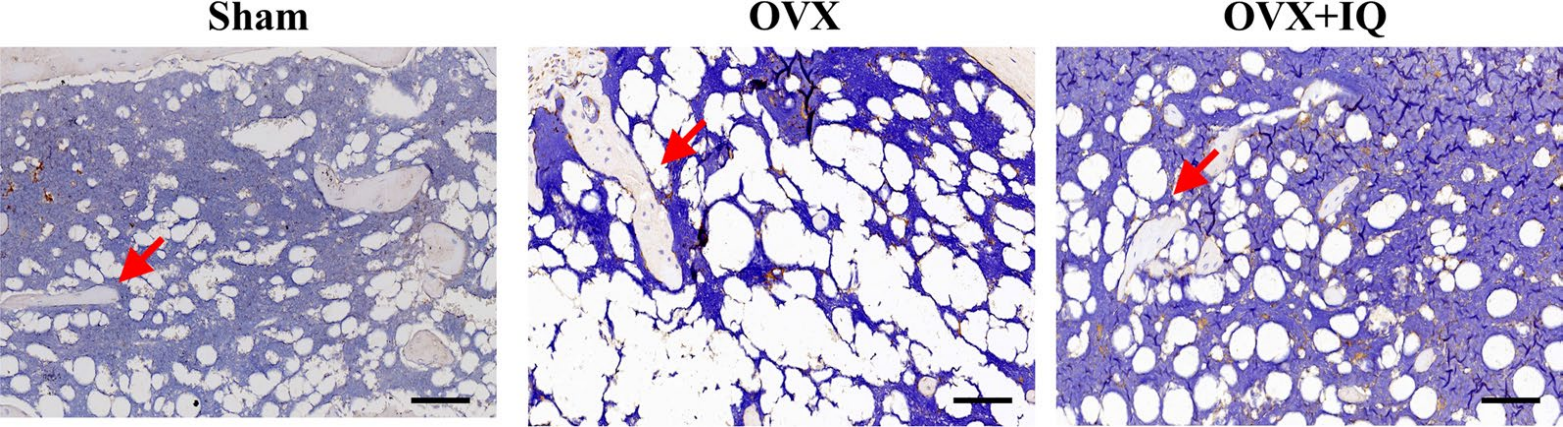
Representative images of IHC staining for detecting the osteocalcin (OCN) expression reflecting the osteoblast activity (scale bar, 100 μm)

Osteocalcin (OCN) is synthesized and secreted by mature osteoblasts (OBs) and is considered as one of the differentiation markers of OBs toward mineralization. Immunostaining results show a significant loss of OCN-positive osteoblasts in OVX mice, while the decrease in the number of osteoblasts is alleviated when OVX mice are treated with genistein. Moreover, slice data reveals that OVX mice exhibit an increase in trabecular space and appear porous. Please refer to Fig. 5 for details.

## Experimental section

### Cell viability assay

Isoquercetin (quercetin-3-glucoside) with 98% purity, was obtained from Golden Health Biotechnology Co., Ltd. (Guangdong, China) and named as BioIQC. C3H10T1/2 cells were seeded at a density of approximately 3000–5000 cells per well in a 96-well plate. After the cells were fully covered in the plate, they were treated with different concentrations (0, 0.01, 0.1, 1, 10, 100 μmol/L) of genistein for 48 h. First, the culture medium was replaced with fresh medium, and then a cell counting kit-8 assay (CK-04, Dojindo) reagent was added and incubated in a cell incubator for 1 h. The absorbance was detected at a wavelength of 450 nm using an enzyme-linked immunosorbent assay reader, and the experiment was repeated twice.

### Cell induction of C3H10T1/2 cells

C3H10T1/2 cells were seeded in a 12-well plate for osteogenic or adipogenic induction. Osteogenic induction was performed with complete culture medium α-MEM (41500-034, Gibco) (containing 10 mmol/L β-glycerophosphate (G9422, Sigma), 50 μmol/L ascorbic acid phosphate (A4403, Sigma), and 0.1 μmol/L dexamethasone (D4902, Sigma)). Adipogenic induction was performed with complete culture medium DMEM (containing 1 μL/mL dexamethasone (D4902, Sigma), 10 μg/mL insulin (I3536, Sigma), 0.5 mmol/L IBMX (I8450, Solarbio), and 200 μmol/L indomethacin (II0100, Solarbio)). The cells were treated with different concentrations (0, 0.01, 0.1, 1, 10, 100 μmol/L) of genistein for 14 days, and the culture medium was replaced every other day.

### Alizarin red and alkaline phosphatase staining

After 14 days of osteogenic differentiation, C3H10T1/2 cells were fixed with 4% paraformaldehyde solution, washed with PBS, and stained with BCIP/NBT alkaline phosphatase staining kit (C3206, Beyotime) and alizarin red solution (G1452, Solarbio). The cells were then washed with distilled water to stop the reaction.

### Nile red staining

Nile red staining (19123, Sigma) solution was prepared by dissolving Nile red in acetone at a concentration of 0.1 mg/mL. The solution was diluted with PBS at a ratio of 1:200 and added to induced adipocytes. The stained adipocytes were then observed under a fluorescence microscope.

### Quantitative real-time polymerase chain reaction analysis (QPCR)

Real-time fluorescent quantitative PCR analysis was performed using a 6-well plate of induced cells collected and total RNA extracted using RNAiso Plus reagent (9109, TaKaRa), followed by reverse transcription using a reverse transcription kit. We applied the PowerUp SYBR Green maffilester mix for real-time fluorescent quantitative PCR on the ABI StepOne real-time fluorescent quantitative PCR system (Applied Biosystems). The PCR cycle parameters were set according to the system's specifications.

Primers used for quantitative real-time PCR

**Table:** 

	Forward primer (5′-3′)	Reverse primer (5′-3′)
*Gapdh*	AAATGGTGAAGGTCGGTGTGAAC	CAACAATCTCCACTTTGCCACTG
*Alpl*	TGACCTTCTCTCCTCCATCC	CTTCCTGGGAGTCTCATCCT
*Runx2*	AGGGACTATGGCGTCAAACA	GGCTCACGTCGCTCACTT
*Il-6*	TTCCATCCAAGTTGCCTTCTTG	TTGGGAGTGGTATCCTCTGTGA
*Occludin*	CCTCCAATGGCAAAGTGAAT	CTCCCCACCTGTCGTGTAGT
*Febp4*	AAGGTGAAGAGCATCATAACCCT	TCACGCCTTTCATAACACATTCC
*Pparγ*	CACCAGTGTGAATTACAGCAAATC	ACAGGAGAATCTCCCAGAGTTTC
*Cebpa*	CAAGAACAGCAACGAGTACCG	GTCACTGGTCAACTCCAGCAC

### Animal experiments

Animal experiments were reviewed and approved by the animal ethics committee of the Innovation Academy for Precision Measurement Science and Technology, Chinese Academy of Sciences (APM20008A). C57BL/6J female mice (6-week-old, 16–18 g) were obtained from Charles River Laboratories (Charles River Co. Ltd, Beijing, China). The mice were housed at the SPF animal facility of the Wuhan Institute of Precision Measurement of the Chinese Academy of Sciences, with a humidity range of 40–60%, and alternating periods of light and dark for 12 h each. The mice were allowed free access to water and food. Following an adaptation period, the mice were randomly assigned to one of three groups: The sham surgery group, OVX model group, or IQ treatment group. The mice in the IQ treatment group received feed containing 0.2% isoquercetin by oral gavage, while the blank control group and the model group received regular maintenance feed. Diet was changed weekly, and mouse’s body weight was recorded every week. Isoquercetin-containing feed was offered to start from Week 0, while OVX surgery was performed in Week 2. Mice were sacrificed at Week 10, and femurs and tibias were collected for further analysis.

### Statistical data analysis

Statistical data analyses were performed using Graph-Pad Prism 8.0 software and presented as the mean ± SD. P values were determined by one-way ANOVA with multiple comparisons and a two-tailed Student’s t-test was performed between two groups. p < 0.05 was considered as statistical significance.

## Conclusions

The process of bone reconstruction is achieved via the collaborative efforts of osteoblasts and osteoclasts. Mesenchymal stem cells in bone marrow differentiate into osteoblasts, making it vital to promote their differentiation to treat osteoporosis effectively. It is noteworthy that mesenchymal stem cells have the potential to transform into adipocytes, not just osteoblasts. In the advanced stage of osteoporosis, insufficient differentiation towards osteoblasts and excessive differentiation towards adipocytes is responsible for the condition. Therefore, promoting osteoblast differentiation in mesenchymal stem cells is essential to treat osteoporosis.

This study investigated the effects of isoquercetin on the proliferation, differentiation, and mineralization of C3H10T1/2 cells, and revealed the mechanism in promoting osteoblast differentiation. The results showed that isoquercetin effectively promoted osteogenic differentiation from mesenchymal stem cells in bone marrow, enhancing the expression of osteogenic differentiation markers *Alpl, Runx2, and Ocn*. The study further explored the effect of isoquercetin on the adipogenic differentiation of mesenchymal stem cells. Isoquercetin was found to significantly inhibit the formation of lipid droplets in adipocytes, as evidenced by fluorescent staining, while qPCR detection demonstrated that isoquercetin could inhibit the expression of adipogenic differentiation markers Pparγ, Fabp4, and Cebpa. Overall, the findings indicate that isoquercetin has considerable potential to promote the differentiation of mesenchymal stem cells towards osteoblasts, as well as inhibiting differentiation towards adipocytes. Significantly, the study established an osteoporosis mouse model to simulate the pathological and physiological state of postmenopausal women with osteoporosis. With the use of imaging and histology, it has been confirmed that isoquercetin had the ability to alleviate bone loss in OVX mice and enhanced the microstructure of bone trabeculae. These findings highlight the potential of isoquercetin as a natural medicine for postmenopausal osteoporosis, paving the way for further research and clinical development. 

## Data Availability

The data supporting the findings of this study are available upon reasonable request from the corresponding author.
